# Dichlorido(6-methyl-2,2′-bipyridine-κ^2^ *N*,*N*′)cobalt(II)

**DOI:** 10.1107/S1600536812041116

**Published:** 2012-10-06

**Authors:** Niloufar Akbarzadeh Torbati, Ali Reza Rezvani, Hamideh Saravani

**Affiliations:** a Department of Chemistry, University of Sistan and Baluchestan, PO Box 98135-674, Zahedan, Iran

## Abstract

In the title compound, [CoCl_2_(C_11_H_10_N_2_)], the Co^II^ atom is four-coordinated in a distorted tetra­hedral geometry by two N atoms from a 6-methyl-2,2′-bipyridine ligand and two terminal Cl atoms. Inter­molecular C—H⋯Cl hydrogen bonds and π–π stacking inter­actions between the pyridine rings [centroid–centroid distance = 3.745 (3) Å] are present in the crystal.

## Related literature

For related structures, see: Ahmadi *et al.* (2009 ▶); Ahmadi, Ebadi *et al.* (2008 ▶); Ahmadi, Kalateh *et al.* (2008 ▶); Akbarzadeh Torbati *et al.* (2010*a* ▶,*b* ▶, 2011 ▶); Amani *et al.* (2009 ▶); Kalateh *et al.* (2010 ▶); Newkome *et al.* (1982 ▶); Onggo *et al.* (1990 ▶, 2005 ▶); Shirvan & Haydari Dezfuli (2012 ▶).
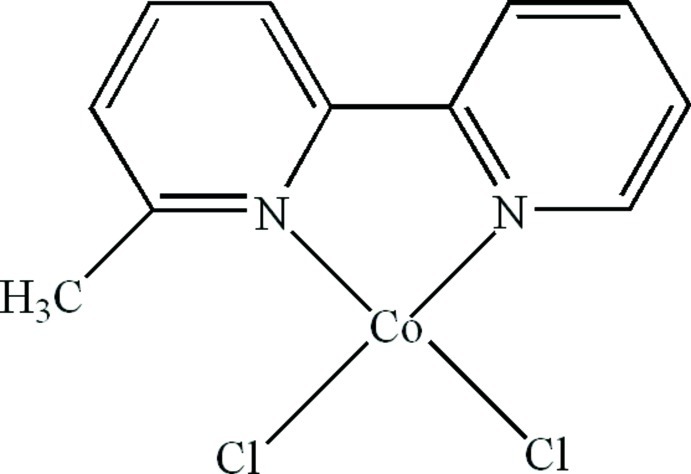


## Experimental

### 

#### Crystal data

[CoCl_2_(C_11_H_10_N_2_)]
*M* *_r_* = 300.04
Monoclinic, 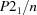

*a* = 7.4395 (6) Å
*b* = 9.4723 (8) Å
*c* = 17.6439 (15) Å
β = 96.131 (7)°
*V* = 1236.24 (18) Å^3^
*Z* = 4
Mo *K*α radiation
μ = 1.79 mm^−1^
*T* = 298 K
0.20 × 0.15 × 0.14 mm

#### Data collection

Bruker APEXII CCD diffractometer
Absorption correction: multi-scan (*SADABS*; Bruker, 2001 ▶) *T*_min_ = 0.730, *T*_max_ = 0.780
7959 measured reflections
3305 independent reflections
2481 reflections with *I* > 2σ(*I*)
*R*_int_ = 0.043

#### Refinement

*R*[*F* ^2^ > 2σ(*F* ^2^)] = 0.058
*wR*(*F* ^2^) = 0.131
*S* = 1.12
3305 reflections
145 parameters
H-atom parameters constrained
Δρ_max_ = 0.85 e Å^−3^
Δρ_min_ = −0.48 e Å^−3^

### 

Data collection: *APEX2* (Bruker, 2007 ▶); cell refinement: *SAINT* (Bruker, 2007 ▶); data reduction: *SAINT*; program(s) used to solve structure: *SHELXS97* (Sheldrick, 2008 ▶); program(s) used to refine structure: *SHELXL97* (Sheldrick, 2008 ▶); molecular graphics: *ORTEP-3* (Farrugia, 1997 ▶) and *Mercury* (Macrae *et al.*, 2006 ▶); software used to prepare material for publication: *SHELXTL* (Sheldrick, 2008 ▶).

## Supplementary Material

Crystal structure: contains datablock(s) I. DOI: 10.1107/S1600536812041116/hy2591sup1.cif

Structure factors: contains datablock(s) I. DOI: 10.1107/S1600536812041116/hy2591Isup2.hkl

Additional supplementary materials:  crystallographic information; 3D view; checkCIF report

## Figures and Tables

**Table 1:** Selected bond lengths (Å)

Co1—N1	2.034 (3)
Co1—N2	2.038 (3)
Co1—Cl1	2.2320 (13)
Co1—Cl2	2.2121 (11)

**Table 2:** Hydrogen-bond geometry (Å, °)

*D*—H⋯*A*	*D*—H	H⋯*A*	*D*⋯*A*	*D*—H⋯*A*
C1—H1*C*⋯Cl1^i^	0.96	2.78	3.706 (6)	163

Symmetry code: (i) 
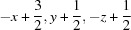
.

**Fig. 1.**
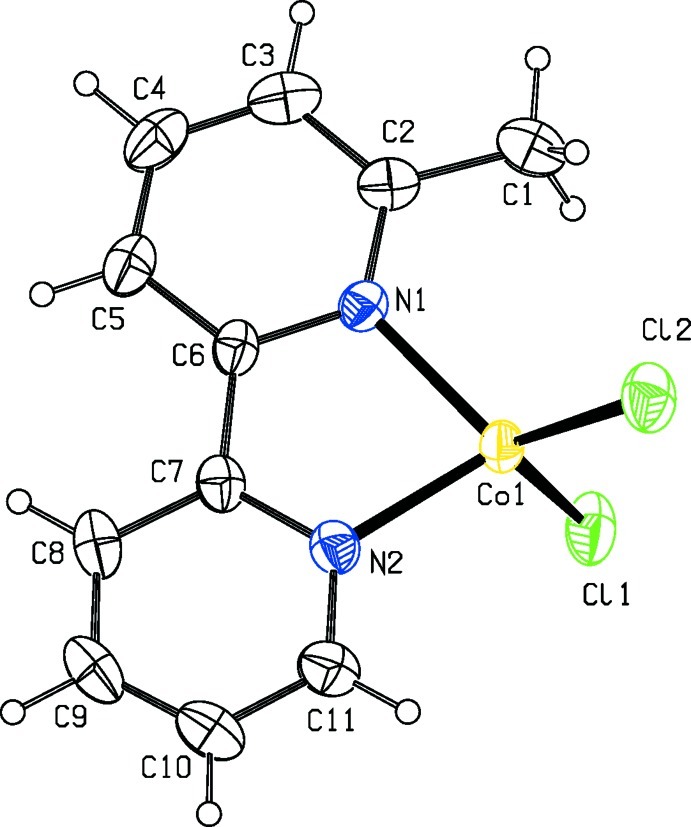
The molecular structure of the title compound. Displacement ellipsoids are drawn at the 30% probability level.

**Fig. 2.**
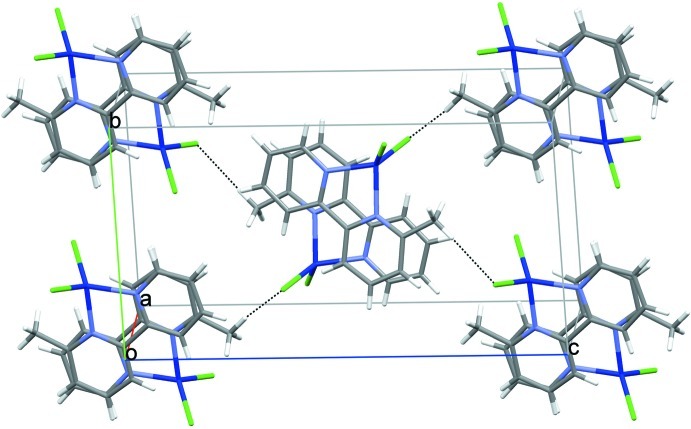
A packing diagram of the title compound. Hydrogen bonds are shown as dashed lines.

## supplementary crystallographic information

### Comment

In recent years, we have reported the synthesis and crystal structures of [Co(6,6'-dmbipy)Cl_2_], (II) (Akbarzadeh Torbati *et al.*, 2010*b*), [Co(Ph_2_phen)Cl_2_], (III) (Akbarzadeh Torbati *et al.*, 2010*a*) and [Co(biq)Cl_2_], (IV) (Akbarzadeh Torbati *et al.*, 2011) (6,6'-dmbipy = 6,6'-dimethyl-2,2'-bipyridine, Ph_2_phen = 2,9-dimethyl-1,10-phenanthroline, biq = 2,2'-biquinoline). 6-Methyl-2,2'-bipyridine (6-mbipy) is a good ligand and a few complexes with 6-mbipy have been prepared, such as that of [Hg(6-mbipy)Cl_2_], (V) (Ahmadi, Ebadi *et al.*, 2008), [Pt(6-mbipy)Cl_4_], (VI) (Amani *et al.*, 2009), [Pb_4_(NO_3_)_8_(6-mbipy)_4_], (VII) (Ahmadi *et al.*, 2009), [Zn(6-mbipy)Br_2_], (VIII) (Kalateh *et al.*, 2010), [Zn(6-mbipy)Cl_2_], (IX) (Ahmadi, Kalateh *et al.*, 2008), [Pd(6-mbipy)Cl_2_], (X) (Newkome *et al.*, 1982), [Ru(6-mbipy)_3_][BF_4_]_2_, (XI) (Onggo *et al.*, 2005), [Fe(6-mbipy)_3_][ClO_4_]_2_.6-mbipy, (XII) (Onggo *et al.*, 1990) and [Cd(6-mbipy)Br_2_(DMSO)], (XIII) (Shirvan & Haydari Dezfuli, 2012). We report herein the synthesis and crystal structure of the title compound, (I).

In the title compound (Fig. 1), the Co^II^ atom is four-coordinated in a distorted tetrahedral geometry by two N atoms from a 6-methyl-2,2'-bipyridine ligand and two terminal Cl atoms (Table 1). In the crystal, intermolecular C—H···Cl hydrogen bonds (Table 2) and π–π contacts (Fig. 2) between the pyridine rings, *Cg*2···*Cg*3^i^ [centroid–centroid distance = 3.745 (3) Å, symmetry code: (i) 2-x, 2-y, -z, *Cg*2 and *Cg*3 are the centroids of the N1/C2–C6 ring and N2/C7–C11 ring, respectively], stabilize the structure.

### Experimental

For the preparation of the title compound, a solution of 6-methyl-2,2'-bipyridine (0.23 g, 1.34 mmol) in methanol (15 ml) was added to a solution of CoCl_2_.6H_2_O (0.37 g, 1.34 mmol) in acetonitrile (15 ml) and the resulting blue solution was stirred for 15 min at 313 K. This solution was left to evaporate slowly at room temperature. After one week, blue block crystals of the title compound were isolated (yield: 0.30 g, 74.6%).

### Refinement

H atoms were positioned geometrically and refined as riding atoms, with C—H = 0.93 (aromatic) and 0.96 (CH_3_) Å and with *U*_iso_(H) = 1.2*U*_eq_(C).

### Crystal data

**Table:**

[CoCl_2_(C_11_H_10_N_2_)]	*F*(000) = 604
*M**_r_* = 300.04	*D*_x_ = 1.612 Mg m^−^^3^
Monoclinic, *P*2_1_/*n*	Mo *K*α radiation, λ = 0.71073 Å
Hall symbol: -P 2yn	Cell parameters from 998 reflections
*a* = 7.4395 (6) Å	θ = 2.3–29.3°
*b* = 9.4723 (8) Å	µ = 1.79 mm^−^^1^
*c* = 17.6439 (15) Å	*T* = 298 K
β = 96.131 (7)°	Block, blue
*V* = 1236.24 (18) Å^3^	0.20 × 0.15 × 0.14 mm
*Z* = 4	

### Data collection

**Table:**

Bruker APEXII CCD diffractometer	3305 independent reflections
Radiation source: fine-focus sealed tube	2481 reflections with *I* > 2σ(*I*)
Graphite monochromator	*R*_int_ = 0.043
φ and ω scans	θ_max_ = 29.3°, θ_min_ = 2.3°
Absorption correction: multi-scan (*SADABS*; Bruker, 2001)	*h* = −10→10
*T*_min_ = 0.730, *T*_max_ = 0.780	*k* = −11→12
7959 measured reflections	*l* = −24→23

### Refinement

**Table:**

Refinement on *F*^2^	Primary atom site location: structure-invariant direct methods
Least-squares matrix: full	Secondary atom site location: difference Fourier map
*R*[*F*^2^ > 2σ(*F*^2^)] = 0.058	Hydrogen site location: inferred from neighbouring sites
*wR*(*F*^2^) = 0.131	H-atom parameters constrained
*S* = 1.12	*w* = 1/[σ^2^(*F*_o_^2^) + (0.0444*P*)^2^ + 1.4058*P*] where *P* = (*F*_o_^2^ + 2*F*_c_^2^)/3
3305 reflections	(Δ/σ)_max_ < 0.001
145 parameters	Δρ_max_ = 0.85 e Å^−^^3^
0 restraints	Δρ_min_ = −0.48 e Å^−^^3^

### Special details

**Table:**

Geometry. All e.s.d.'s (except the e.s.d. in the dihedral angle between two l.s. planes) are estimated using the full covariance matrix. The cell e.s.d.'s are taken into account individually in the estimation of e.s.d.'s in distances, angles and torsion angles; correlations between e.s.d.'s in cell parameters are only used when they are defined by crystal symmetry. An approximate (isotropic) treatment of cell e.s.d.'s is used for estimating e.s.d.'s involving l.s. planes.
Refinement. Refinement of *F*^2^ against ALL reflections. The weighted *R*-factor *wR* and goodness of fit *S* are based on *F*^2^, conventional *R*-factors *R* are based on *F*, with *F* set to zero for negative *F*^2^. The threshold expression of *F*^2^ > σ(*F*^2^) is used only for calculating *R*-factors(gt) *etc*. and is not relevant to the choice of reflections for refinement. *R*-factors based on *F*^2^ are statistically about twice as large as those based on *F*, and *R*- factors based on ALL data will be even larger.

### Fractional atomic coordinates and isotropic or equivalent isotropic displacement parameters (Å^2^)

**Table:**

	*x*	*y*	*z*	*U*_iso_*/*U*_eq_	
C1	0.6700 (9)	0.9546 (6)	0.2127 (3)	0.0841 (16)	
H1A	0.7822	0.9086	0.2296	0.101*	
H1B	0.5749	0.8858	0.2064	0.101*	
H1C	0.6430	1.0233	0.2498	0.101*	
C2	0.6857 (6)	1.0262 (5)	0.1385 (3)	0.0549 (10)	
C3	0.6639 (6)	1.1710 (5)	0.1282 (3)	0.0661 (12)	
H3	0.6359	1.2279	0.1683	0.079*	
C4	0.6843 (7)	1.2286 (5)	0.0589 (3)	0.0679 (13)	
H4	0.6706	1.3254	0.0518	0.081*	
C5	0.7246 (6)	1.1457 (4)	−0.0005 (3)	0.0572 (10)	
H5	0.7389	1.1850	−0.0478	0.069*	
C6	0.7439 (4)	1.0016 (4)	0.0116 (2)	0.0430 (8)	
C7	0.7826 (5)	0.8999 (4)	−0.0480 (2)	0.0446 (8)	
C8	0.8106 (6)	0.9397 (5)	−0.1220 (2)	0.0582 (11)	
H8	0.8110	1.0345	−0.1357	0.070*	
C9	0.8380 (7)	0.8356 (6)	−0.1748 (3)	0.0686 (13)	
H9	0.8574	0.8599	−0.2243	0.082*	
C10	0.8363 (7)	0.6973 (6)	−0.1535 (3)	0.0703 (13)	
H10	0.8512	0.6264	−0.1888	0.084*	
C11	0.8125 (6)	0.6635 (5)	−0.0799 (3)	0.0601 (11)	
H11	0.8138	0.5690	−0.0656	0.072*	
N1	0.7245 (4)	0.9445 (3)	0.08066 (17)	0.0431 (7)	
N2	0.7872 (4)	0.7629 (3)	−0.02746 (18)	0.0465 (7)	
Co1	0.76711 (7)	0.73253 (5)	0.08569 (3)	0.04458 (16)	
Cl1	1.03449 (15)	0.68766 (14)	0.15119 (7)	0.0683 (3)	
Cl2	0.54399 (16)	0.59698 (13)	0.11655 (7)	0.0673 (3)	

### Atomic displacement parameters (Å^2^)

**Table:**

	*U*^11^	*U*^22^	*U*^33^	*U*^12^	*U*^13^	*U*^23^
C1	0.114 (5)	0.085 (4)	0.056 (3)	0.010 (3)	0.024 (3)	−0.014 (3)
C2	0.049 (2)	0.055 (2)	0.061 (2)	0.0011 (18)	0.0059 (18)	−0.0083 (19)
C3	0.056 (2)	0.061 (3)	0.081 (3)	0.005 (2)	0.005 (2)	−0.021 (2)
C4	0.060 (3)	0.040 (2)	0.103 (4)	−0.0006 (19)	0.004 (3)	0.003 (2)
C5	0.052 (2)	0.046 (2)	0.073 (3)	−0.0005 (17)	0.003 (2)	0.013 (2)
C6	0.0343 (16)	0.0436 (18)	0.050 (2)	−0.0035 (14)	0.0007 (14)	0.0110 (15)
C7	0.0353 (16)	0.050 (2)	0.048 (2)	−0.0082 (15)	0.0024 (14)	0.0068 (16)
C8	0.054 (2)	0.070 (3)	0.049 (2)	−0.012 (2)	0.0021 (18)	0.018 (2)
C9	0.066 (3)	0.102 (4)	0.039 (2)	−0.010 (3)	0.0119 (19)	0.003 (2)
C10	0.073 (3)	0.090 (4)	0.050 (3)	−0.005 (3)	0.015 (2)	−0.015 (2)
C11	0.061 (2)	0.063 (3)	0.057 (3)	−0.003 (2)	0.012 (2)	−0.009 (2)
N1	0.0412 (15)	0.0412 (15)	0.0465 (17)	0.0016 (12)	0.0027 (13)	0.0022 (13)
N2	0.0442 (15)	0.0499 (17)	0.0458 (16)	−0.0021 (13)	0.0070 (13)	0.0040 (14)
Co1	0.0474 (3)	0.0416 (3)	0.0458 (3)	0.0009 (2)	0.0098 (2)	0.0099 (2)
Cl1	0.0500 (5)	0.0788 (8)	0.0754 (7)	0.0019 (5)	0.0033 (5)	0.0350 (6)
Cl2	0.0590 (6)	0.0654 (7)	0.0786 (8)	−0.0129 (5)	0.0130 (5)	0.0186 (6)

### Geometric parameters (Å, º)

**Table:**

C1—C2	1.490 (7)	C7—N2	1.346 (5)
C1—H1A	0.9600	C7—C8	1.397 (5)
C1—H1B	0.9600	C8—C9	1.386 (7)
C1—H1C	0.9600	C8—H8	0.9300
C2—N1	1.336 (5)	C9—C10	1.363 (8)
C2—C3	1.391 (6)	C9—H9	0.9300
C3—C4	1.363 (7)	C10—C11	1.367 (6)
C3—H3	0.9300	C10—H10	0.9300
C4—C5	1.368 (7)	C11—N2	1.347 (5)
C4—H4	0.9300	C11—H11	0.9300
C5—C6	1.386 (5)	Co1—N1	2.034 (3)
C5—H5	0.9300	Co1—N2	2.038 (3)
C6—N1	1.355 (5)	Co1—Cl1	2.2320 (13)
C6—C7	1.477 (5)	Co1—Cl2	2.2121 (11)
			
C2—C1—H1A	109.5	C9—C8—C7	118.9 (4)
C2—C1—H1B	109.5	C9—C8—H8	120.6
H1A—C1—H1B	109.5	C7—C8—H8	120.6
C2—C1—H1C	109.5	C10—C9—C8	119.5 (4)
H1A—C1—H1C	109.5	C10—C9—H9	120.2
H1B—C1—H1C	109.5	C8—C9—H9	120.2
N1—C2—C3	120.2 (4)	C9—C10—C11	119.5 (5)
N1—C2—C1	116.8 (4)	C9—C10—H10	120.3
C3—C2—C1	122.9 (4)	C11—C10—H10	120.3
C4—C3—C2	119.3 (4)	N2—C11—C10	122.1 (5)
C4—C3—H3	120.4	N2—C11—H11	119.0
C2—C3—H3	120.4	C10—C11—H11	119.0
C3—C4—C5	120.8 (4)	C2—N1—C6	120.6 (3)
C3—C4—H4	119.6	C2—N1—Co1	125.7 (3)
C5—C4—H4	119.6	C6—N1—Co1	113.7 (2)
C4—C5—C6	118.4 (4)	C7—N2—C11	119.3 (3)
C4—C5—H5	120.8	C7—N2—Co1	113.4 (3)
C6—C5—H5	120.8	C11—N2—Co1	127.2 (3)
N1—C6—C5	120.7 (4)	N1—Co1—N2	81.08 (13)
N1—C6—C7	115.3 (3)	N1—Co1—Cl2	117.79 (9)
C5—C6—C7	123.9 (4)	N2—Co1—Cl2	117.29 (10)
N2—C7—C8	120.7 (4)	N1—Co1—Cl1	109.69 (10)
N2—C7—C6	116.0 (3)	N2—Co1—Cl1	112.31 (10)
C8—C7—C6	123.3 (4)	Cl2—Co1—Cl1	114.38 (5)
			
N1—C2—C3—C4	−0.6 (7)	C5—C6—N1—Co1	−178.0 (3)
C1—C2—C3—C4	178.6 (5)	C7—C6—N1—Co1	3.0 (4)
C2—C3—C4—C5	0.4 (7)	C8—C7—N2—C11	−2.5 (6)
C3—C4—C5—C6	0.2 (7)	C6—C7—N2—C11	176.6 (3)
C4—C5—C6—N1	−0.5 (6)	C8—C7—N2—Co1	174.3 (3)
C4—C5—C6—C7	178.4 (4)	C6—C7—N2—Co1	−6.6 (4)
N1—C6—C7—N2	2.4 (5)	C10—C11—N2—C7	0.9 (7)
C5—C6—C7—N2	−176.5 (4)	C10—C11—N2—Co1	−175.4 (4)
N1—C6—C7—C8	−178.5 (3)	C2—N1—Co1—N2	176.8 (3)
C5—C6—C7—C8	2.6 (6)	C6—N1—Co1—N2	−5.0 (2)
N2—C7—C8—C9	1.9 (6)	C2—N1—Co1—Cl2	60.6 (3)
C6—C7—C8—C9	−177.1 (4)	C6—N1—Co1—Cl2	−121.3 (2)
C7—C8—C9—C10	0.2 (7)	C2—N1—Co1—Cl1	−72.6 (3)
C8—C9—C10—C11	−1.8 (8)	C6—N1—Co1—Cl1	105.6 (2)
C9—C10—C11—N2	1.2 (8)	C7—N2—Co1—N1	6.3 (3)
C3—C2—N1—C6	0.3 (6)	C11—N2—Co1—N1	−177.1 (4)
C1—C2—N1—C6	−178.9 (4)	C7—N2—Co1—Cl2	123.1 (2)
C3—C2—N1—Co1	178.3 (3)	C11—N2—Co1—Cl2	−60.4 (4)
C1—C2—N1—Co1	−0.9 (6)	C7—N2—Co1—Cl1	−101.4 (3)
C5—C6—N1—C2	0.3 (5)	C11—N2—Co1—Cl1	75.2 (4)
C7—C6—N1—C2	−178.7 (3)		

### Hydrogen-bond geometry (Å, º)

**Table:**

*D*—H···*A*	*D*—H	H···*A*	*D*···*A*	*D*—H···*A*
C1—H1*C*···Cl1^i^	0.96	2.78	3.706 (6)	163

Symmetry code: (i) −*x*+3/2, *y*+1/2, −*z*+1/2.
